# Developmental Roles of AUX1/LAX Auxin Influx Carriers in Plants

**DOI:** 10.3389/fpls.2019.01306

**Published:** 2019-10-28

**Authors:** Ranjan Swarup, Rahul Bhosale

**Affiliations:** ^1^Plant and Crop Sciences, School of Biosciences, University of Nottingham, Nottingham, United Kingdom; ^2^Center for Plant Integrative Biology (CPIB), University of Nottingham, Nottingham, United Kingdom

**Keywords:** auxin, AUX1/LAX, influx carriers, plant, development

## Abstract

Plant hormone auxin regulates several aspects of plant growth and development. Auxin is predominantly synthesized in the shoot apex and developing leaf primordia and from there it is transported to the target tissues e.g. roots. Auxin transport is polar in nature and is carrier-mediated. AUXIN1/LIKE-AUX1 (AUX1/LAX) family members are the major auxin influx carriers whereas PIN-FORMED (PIN) family and some members of the P-GLYCOPROTEIN/ATP-BINDING CASSETTE B4 (PGP/ABCB) family are major auxin efflux carriers. AUX1/LAX auxin influx carriers are multi-membrane spanning transmembrane proteins sharing similarity to amino acid permeases. Mutations in *AUX1/LAX* genes result in auxin related developmental defects and have been implicated in regulating key plant processes including root and lateral root development, root gravitropism, root hair development, vascular patterning, seed germination, apical hook formation, leaf morphogenesis, phyllotactic patterning, female gametophyte development and embryo development. Recently AUX1 has also been implicated in regulating plant responses to abiotic stresses. This review summarizes our current understanding of the developmental roles of *AUX1/LAX* gene family and will also briefly discuss the modelling approaches that are providing new insight into the role of auxin transport in plant development.

## Introduction

Auxin is a key plant hormone that regulates several aspects of plant growth and development including plant tropic responses, embryo development, root development, shoot development, leaf development and phylotactic patterning and there are several good reviews highlighting the role of auxin in plant development (Reinhardt, 2003; Leyser, 2006; Sieburth and Deyholos, 2006; Vanneste and Friml, 2009; Wang and Estelle, 2014; Lavy and Estelle, 2016).

It is known for a long time that auxin transport is polar in nature. Following its synthesis in the leaf primordia and shoot apical meristem, auxin is transported downwards to its target tissues using either through the bulk flow in the phloem or through the polar auxin transport stream (Swarup et al., 2001; Vanneste and Friml, 2009; Swarup and Péret, 2012; Swarup and Bennett, 2014).

Auxin was the first plant hormone to be discovered (Went, 1927). Indole-3-acetic acid (IAA), the major form of auxin in higher plants, is a weak acid (pKa = 4.85) and, at the intracellular pH, exists in its membrane impermeable (IAA-) form. However, in the extracellular apoplast, where the pH is slightly acidic (pH ∼5.5), IAA exists both as membrane permeable IAAH form and membrane impermeable (IAA-) form (Zazímalová et al., 2010; Swarup and Péret, 2012; Swarup and Bennett, 2014). Zazímalová et al. (2010) highlighted the importance of auxin influx carriers when they calculated that at apoplastic pH, 83% of IAA is in its membrane impermeable IAA- form and would need a carrier for its import in the cell. Interestingly, even before any of the auxin carriers were discovered, as part of the chemiosmotic hypothesis, Rubery and Sheldrake (1974) and Raven (1975) independently proposed that auxin transport is carrier-mediated and the polarity of auxin movement is likely to be provided by asymmetric localization of auxin transporters (Swarup and Bennett, 2014; Mohanta et al., 2018). It is now well established that auxin transport is carrier-mediated and is facilitated by auxin influx carriers and efflux carriers. Auxin influx carriers mediate the uptake of auxin inside the cells whereas auxin efflux carriers are required for the export of auxin out of the plant cells. In *Arabidopsis*, the AUX1/LAX family of auxin transporters represent the major influx carriers (Bennett et al., 1996; Swarup et al., 2001; Swarup et al., 2008; Péret et al., 2012) whereas PIN (Chen et al., 1998; Gälweiler et al., 1998; Luschnig et al., 1998; Steinmann et al., 1999; Friml et al., 2002; Friml et al., 2003; Blilou et al., 2005; Wisniewska et al., 2006; Rahman et al., 2010; Bosco et al., 2012) and PGP/ABCB (Noh et al., 2003; Terasaka et al., 2005; Blakeslee et al., 2007; Cho et al., 2007; Zhang et al., 2018) family members encode the major auxin efflux carriers. Using an *in-silico* approach, Barbez et al. (2012) identified *PILS* (*PIN LIKES*) gene family that also appears to be regulating auxin homeostasis. In addition, it has been suggested that nitrate transporter NRT1.1 can also be involved in auxin transport (Krouk et al., 2010) and can regulate lateral root formation depending on the nitrogen status of the plant (Krouk et al., 2010). This may provide a direct mechanism for soil nutrient status mediated auxin dependent regulation of lateral root development.

This review will focus on our current understanding of the roles of AUX1/LAX proteins in regulating auxin transport during plant development. There have been a few comprehensive reviews covering the role of auxin influx carriers in plant development in general (Swarup and Péret, 2012; Swarup and Bennett, 2014) or AUX1 in particular (Singh et al., 2018) and so this review will only briefly discuss the topics covered in those reviews. Here, we focus primarily on new understanding of AUX1/LAX auxin influx carriers and their roles in plant development.

## AUX1/LAX Gene Family in *Arabidopsis*

In the chemiosmotic hypothesis, both Rubery and Sheldrake (1974) and Raven (1975) independently proposed that auxin transport is carrier-mediated but it was not until 1985 when Lomax et al. (1985) showed that IAA uptake is an active process and is driven by proton motive force.

Working on suspension-cultured tobacco cells, Delbarre et al. (1996) revealed that IAA and 2,4-D (2,4-Dichlorophenoxyacetic acid-a synthetic auxin) uptake in the cells is carrier-mediated but in contrast, lipophilic auxin 1-naphthalene acetic acid (1-NAA) enters the cells through passive diffusion. This indeed turned out to be true when the first auxin influx carrier was cloned and characterized (Bennett et al., 1996; Marchant et al., 1999). Cloning of *AUX1* gene revealed similarity to amino acid permeases. Considering IAA is structurally similar to tryptophan, explains the evolution of these plant specific sub class of the amino acid permease superfamily that now is known as auxin amino acid permease superfamily. In *Arabidopsis*, the *AUX1/LAX* gene family is comprised of four members *AUX1, LAX1, LAX2*, and *LAX3* sharing 75–80% similarity at protein level (Swarup and Péret, 2012; Swarup and Bennett, 2014). These genes encode multi-transmembrane (TM) spanning proteins and share similarity to amino acid transporters (Young et al., 1999; Péret et al., 2012). *Arabidopsis* AUX1/LAX proteins have been shown to take up auxin in heterologous expression systems (Yang et al., 2006; Swarup et al., 2008; Péret et al., 2012) and mutations in *AUX1/LAX* genes result in auxin related developmental defects (Bennett et al., 1996; Swarup et al., 2001; Swarup et al., 2004; Swarup et al., 2005; Swarup et al., 2007; Swarup et al., 2008; Bainbridge et al., 2008; Péret et al., 2012). The founding member of this family, AUX1 has been well studied and shown to regulate root gravitropism (Bennett et al., 1996; Swarup et al., 2001; Swarup et al., 2004; Swarup et al., 2005), whereas AUX1 and LAX3 both shown to regulate lateral root development (Swarup et al., 2008). LAX2 has been shown to facilitate vascular development (Péret et al., 2012) and AUX1, LAX1 and LAX2 have been shown to act in a redundant manner to regulate phyllotactic patterning (Bainbridge et al., 2008). These studies highlight the functional importance of AUX1/LAX proteins in auxin transport.

**Figure 1:**
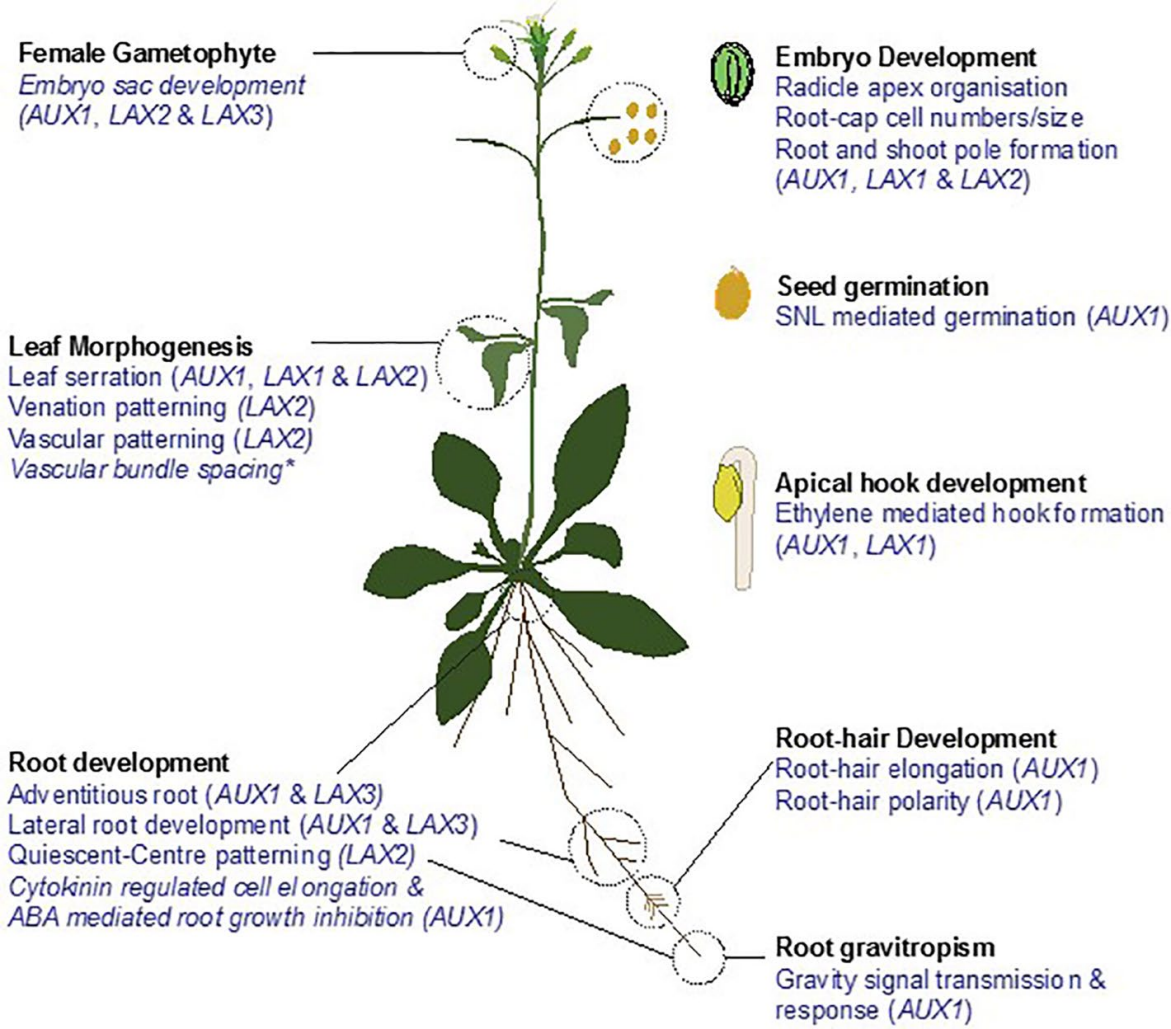
Roles of AUX1/LAX family in development of *Arabidopsis thaliana*. Auxin influx carrier *AUX1* and its homologs *LAX1*, *LAX2*, and *LAX3* have been implicated in regulating root gravitropism; root, lateral & adventitious root development; root-hair development, leaf morphogenesis; female gametophyte development; embryo development; seed germination and apical hook development. Names in parenthesis indicate the genes controlling the respective role. *indicates the role of AUX1/LAX family deciphered from modelling studies and quadruple mutant analysis. Aradidopsis plant cartoon template adapted from (Bhosale et al., 2019).

In the following sections, we will review our understanding of the role of AUX1/LAX auxin influx carriers in regulating plant development in *Arabidopsis* and highlight their importance in several biological processes from seed germination to root, shoot, and flower development and embryogenesis (**Figure 1**).

### Root Gravitropism

Gravity in the roots is perceived in the columella cells. Upon gravity perception, differential movement of auxin from the site of gravity perception to the gravity-responsive tissues of the elongation zone results in root bending as higher auxin levels on the lower side of the root inhibits cell elongation whereas cells on the upper side are still elongating (Ottenschläger et al., 2003; Swarup et al., 2013; Sato et al., 2015; Muller et al., 2018). Differential auxin gradient is facilitated by auxin efflux carrier PIN3 that relocalizes to the new bottom of the cell within minutes after the gravity stimulus (Friml et al., 2002). In addition, PIN7 that has been reported to regulate root bending in detached lateral roots (Ruiz Rosquete et al., 2018), appears to be involved in some kind of compensation mechanism in primary roots as PIN7-GFP is expanded into the PIN3 expression domain in the pin3 mutants (Kleine-Vehn et al., 2010). Using a modelling approach, Band et al. (2012), revealed that auxin asymmetry is transient and lasts only about 100 min and after which the auxin distribution becomes symmetric. They proposed a “tipping point mechanism” that reverses the auxin flow as the root tips reach an angle of 40º.

Among the AUX1/LAX family members in *Arabidopsis*, only AUX1 has been implicated in regulating root gravitropic response. A recent report suggests that AUX1 acts upstream of PIN2 in regulating root gravitropism (Liu et al., 2018) but we believe that they act in concert. Mutation in *aux1* results in agravitropic roots. *AUX1* is expressed in tissues involved in gravity perception (columella), gravity signal transmission (lateral root cap) and gravity response (epidermis) (Swarup et al., 2001; Swarup et al., 2004; Swarup et al., 2005). Using a transactivation-based approach, Swarup et al. (2005) probed which AUX1 expression domains are required for root gravitropism and revealed that *AUX1* expression in lateral root cap and epidermal cells was sufficient to rescue root gravitropic defect of *aux1* mutants. Interestingly, *AUX1* columella expression was not required for restoration of root agravitropic defect of *aux1* mutants. Genetic redundancy could be a possible explanation as both *LAX2* and *LAX3* are known to express in the columella cells.

Recently, Pernisova et al. (2016) proposed that AUX1 mediated auxin influx is subject to regulation by cytokinins. They found that multiple cytokinin receptor mutants show altered root angles in gravi-stimulated roots and *35S:CKX* lines over expressing cytokinin degradation enzyme cytokinin dehydrogenase/oxidase show delayed root gravitropism. Interestingly, treatment with exogenous cytokinins does not affect root gravitropic response and so the authors suggest that proper concentration of endogenous cytokinins is crucial for gravity induced auxin redistribution. They also show that AUX1-YFP signal is reduced in these cytokinin depleted lines but the relocalization kinetics of PIN3 and PIN7 was comparable to wild type. It will be interesting to check if treatment with exogenous cytokinins can restore AUX1 signal in the cytokinin depleted lines to further understand the role of cytokinin in regulating auxin influx and root gravitropism.

### Vascular Development

Auxin is known to regulate vascular development. Direct treatment with auxin promotes vascular development and several auxin transport and response mutants have defects in vascular patterning (Reinhardt, 2003; Sieburth and Deyholos, 2006; Péret et al., 2009a; Péret et al., 2009b; Petrásek and Friml, 2009; Yang and Wang, 2016). Despite, the role of auxin influx carriers has not been very clear until recently. Now, there is some evidence for the role of LAX2 in regulating vascular patterning in the cotyledons as *lax2* mutants have higher propensity of discontinuity in vascular strands in the cotyledon (Péret et al., 2012). More recently, Fàbregas et al. (2015) used a computational, modelling and experimental approaches to investigate the role of influx carriers in vascular development. Their theoretical approach predicted that the auxin influx carriers increase the periodicity of auxin maxima. They tested these predictions using *aux1/lax* quadruple mutants and showed that these mutants have fewer and more spaced vascular bundles. Additionally, they showed that AUX1/LAX proteins also regulate xylem differentiation in both the shoot and the root.

### Seed Germination

Plant hormones such as Gibberellic Acid (GA) and Abscisic Acid (ABA) are known to regulate seed germination (Koornneef et al., 2002; Finkelstein et al., 2008). GA promotes germination and ABA inhibits germination. Auxin has also been implicated in regulating germination (Holdsworth et al., 2008). Low concentration of auxin promotes seed germination and higher concentrations inhibit germination (Hsueh and Lou, 1947; Brady et al., 2003). Auxin appears to affect seed germination through GA/ABA signaling pathways as auxin response factors ARF10 and ARF16 can modulate expression of *ABSCISIC ACID INSENSITIVE 3 (ABI3)* and imbibed seeds have increased expression of auxin influx and efflux carriers compared to dormant seeds (Carrera et al., 2007; Liu et al., 2013).

It is emerging now that histone modification may play a crucial role in regulating seed germination by regulating the expression of several auxin related genes. Recently, Wang et al. (2017) showed that mutations in histone deacetylase-binding factor genes, *SNL1 (SWI-INDEPENDENT3 (SIN3)-LIKE1)* and *SNL2* result in increased speed of seed germination and faster radicle protrusion. They also showed that expression of several auxin synthesis, transport and responses genes were up regulated in *snl1snl2* double mutants and these mutants accumulated significantly more IAA than wild type plants. This led Wang et al. (2017) to investigate the seed germination in auxin related mutants. Though many auxin related genes were upregulated in *snl1snl2* double mutant, many of their mutants had no obvious seed germination defects. However, they did find that *aux1* mutants had weakly decreased germination of fresh seeds compared to controls. Further investigation showed that AUX1 levels were higher in the radicle tip of the *snl1snl2* double mutants and AUX1 was a target for SNL mediated H3K9/18 histone deacetylation. Moreover, they also showed that the cell cycle genes *CYCD1;1* and *CYCD4;1* were upregulated in *snl1snl2* double mutants, which is also in agreement with increased cell cycle activity in these mutants. In addition, auxin also can induce the expression of *CYCD1;1* and *CYCD4;1*. Taken together, they showed that SNLs negatively regulate radicle protrusion and AUX1 plays a crucial role in SNL mediated seed germination. They further proposed a model, where during embryo development and seed maturation high SNL levels cause deacetylation of ABA hydrolysis genes causing high ABA levels inhibiting seed germination. During imbibition, the SNL levels go down that promotes expression of auxin synthesis and transport genes including *AUX1*. Increased auxin levels, in turn, switch on cell cycle genes that promote radicle growth.

### Apical Hook Development

Apical hook protects the shoot apical meristem during seedling emergence from the soil (Abbas et al., 2013). Light and plant hormones such as auxin, ethylene, gibberellins, and brassinosteroids are key signals regulating apical hook development (Abbas et al., 2013; Smet et al., 2014). Vandenbussche et al. (2010) showed that apical hook formation requires fresh auxin synthesis on the inner side of the apical hook in an ethylene dependent fashion. This results in creation of an auxin gradient and the inhibition of cell elongation on the inner side, in turn, resulting in differential growth and apical hook formation.

Among the auxin influx carriers, AUX1 and LAX3 have been implicated in regulating apical hook development in *Arabidopsis* (Roman et al., 1995; Vandenbussche et al., 2010). Vandenbussche et al. (2010) showed that *aux1* and *lax3* mutants show less exaggerated apical hook upon ethylene treatment. *AUX1* is expressed in the hook region and it is localized in the epidermal cell membranes (Vandenbussche et al., 2010). In contrast, *LAX3* is localized to the plasma membrane in the outer tissues on the basal parts (but not in the apical hook region) of hypocotyl. Vandenbussche et al. (2010) proposed that LAX3 is the major auxin influx carrier in hook development with AUX1 facilitating movement of auxin from the cotyledons and shoot apical meristem to the apical hook whereas LAX3 draining auxin out of the hook.

Though both AUX1 and LAX3 (along with auxin efflux carrier PIN3— Zádníková et al., 2010) regulate apical hook formation, their localization to the plasma membrane appears to be under distinct genetic regulation. Working in the *echidna* mutants, Boutté et al. (2013) showed that *echidna* mutants have defects in apical hook formation. They also have defects in differential auxin gradient formation in the apical hook. Boutté et al. (2013) showed that ECHIDNA is predominantly localized to the secretory vesicles at the trans-Golgi network (TGN) and in *echidna* mutants AUX1 trafficking to the plasma membrane is disrupted. In contrast, LAX3 or PIN3 trafficking is only marginally altered in *echidna* mutants. These findings suggest that not only there are distinct mechanisms for the trafficking of auxin influx and efflux carrier proteins to the plasma membrane, even the closely related AUX1 and LAX3 follow distinct trafficking pathways from the trans-Golgi network (TGN) to the plasma membrane in the hypocotyl.

Kleine-Vehn et al. (2006) had shown previously that polar AUX1 and PIN1 trafficking to the plasma membrane in the root protophloem cells follow distinct pathways with PIN1 but not AUX1 trafficking being GNOM dependent. *GNOM* encodes membrane-associated guanine–nucleotide exchange factor on ADP-ribosylation factor G protein (ARF-GEF) (Steinmann et al., 1999). Guanine nucleotide exchange factors are involved in the activation of small GTPases which act as molecular switches in the intracellular signaling pathways (Casanova, 2007; Richter et al., 2014). In *Arabidopsis*, there are eight ARF-GEFs which are grouped into two classes based on their similarity to human big ARF-GEFs, GBF1, and BIG1 (not to be confused with calossin-like protein BIG/DOC/CRM—Gil et al., 2001). There are three GBF1 related members (GNOM, GLN1, GLN2) and five BIG1 related members (BIG1 to BIG5) (Richter et al., 2014). BIG1-4 have been implicated in post Golgi trafficking.

Recently, Jonsson et al. (2017) showed that in hook development, AUX1 trafficking is mediated by ECHIDNA, ARF1, and BIG proteins. They showed that ARF1 and BIG4 colocalize with ECHIDNA in the TGN and *arf1* and *big* mutants have defects in AUX1 trafficking and show hook developmental defects similar to *aux1* mutants. In *echidna* mutants, ARF1 and BIG cannot localize to the TGN whereas in *arf1* and *big* mutants, ECHIDNA localization is perturbed. They proposed that ECHIDNA and BIG facilitate recruitment of ARF1 to the TGN that then facilitates vesicle formation for AUX1 delivery to the plasma membrane, which is a prerequisite for the ethylene mediated hook development.

### Root Development

During root development, cells arise linearly from a group of stem cells surrounding the quiescent center (QC). Stem cells divide asymmetrically to give rise to daughter cells that move away from the stem cell niche and differentiate (Scheres, 2007). Auxin is crucial for specification of the QC and QC is crucial for the maintenance of the stem cell fate. Cytokinin is important for promoting cell differentiation and auxin and cytokinin act antagonistically to regulate root development (Aloni et al., 2006).

Zhang et al. (2013) showed that auxin influx carrier LAX2 plays a role in regulating QC patterning in the roots *via* an auxin–cytokinin module. In *Arabidopsis*, cytokinin signaling is mediated by a multi-step phosphorelay mechanism involving cytokinin receptors AHKs (*Arabidopsis* Histidine Kinases) AHPs (*Arabidopsis* histidine phosphotransfer proteins) and ARRs (*Arabidopsis* response regulators). Upon cytokinins perception, AHKs relay the signal to ARRs *via* AHPs. ARRs act as transcriptional regulators to regulate gene expression (Keshishian and Rashotte, 2015). Zhang et al. (2013) show that cytokinin promotes cell division in the QC and downregulate the expression of several genes including *LAX2* in ARR1 and ARR12 dependent fashion and ARR1 directly binds to *LAX2* gene. Moreover, *lax2* mutants have increased cell division in the QC thus phenocopying cytokinin treatment. Taken together, Zhang et al. (2013) proposed that cytokinin suppresses *lax2* expression to regulate auxin distribution in the root apical meristem.

Cytokinins inhibit cell elongation and cell proliferation. Street et al. (2016) recently showed that cytokinin inhibits root cell elongation *but not cell proliferation* in an AUX1 dependent fashion. Using an innovative screen in *arr1* and *arr12* background to identify novel regulators of cytokinin mediated root development. Two of the enhancers (termed *enhancers of response regulators* or *err*) turned out to have mutations in the *AUX1* gene (Pro371Leu and Gly374Ser) in the extracellular loop between 9th and 10th transmembrane regions suggesting the role of AUX1 in cytokinin mediated root development. Indeed, *aux1* mutants showed reduced root growth inhibition to cytokinin treatment but not *lax1*, *lax2* or *lax3*. They further observed that the expression of the type-B response regulator *ARR10* is auxin and AUX1 dependent and led them to propose that cytokinin and auxin regulate expression of *ARR10* and *AUX1* as part of an auto regulatory circuit.

AUX1 has also been implicated in ABA mediated inhibition of root growth. ABA is known to inhibit root growth at high concentration and recently, Li et al. (2017) showed that this is mediated *via* AUX1 in an ethylene dependent pathway.

### Lateral Root Development

Lateral roots originate from the xylem pole pericycle cells that undergo a series of division to create the lateral root primordia (Dubrovsky et al., 2000; Dubrovsky et al., 2001; Péret et al., 2009a; Lavenus et al., 2013; Du and Scheres, 2018). This new primordium has to penetrate several cell layers before emergence (Swarup et al., 2008; Péret et al., 2009b; Swarup and Péret, 2012). Auxin is one of the key signals regulating lateral root development (Benková et al., 2003; Swarup et al., 2008; Péret et al., 2009a; Swarup and Péret, 2012; Du and Scheres, 2018). Mutations in auxin influx carriers *AUX1* result in about 50% reduction in lateral root numbers (Hobbie and Estelle, 1995; Marchant et al., 2002). Swarup et al. (2008) showed that *lax3* mutants also have 50% reduction in lateral root numbers and *aux1lax3* double mutants have severe reduction in lateral root emergence and show almost no emerged lateral root primordia up to day 14. Both *AUX1* and *LAX3* show contrasting and non-overlapping expression patterns with *AUX1* being expressed in the primordia whereas *LAX3* is completely excluded from the primordia and is expressed in the cortical and epidermal cells facing the primordia (Marchant et al., 2002; Swarup et al., 2008). To explain lateral root emergence, Swarup et al. (2008) proposed an elegant model based on the facts that the *LAX3* gene is auxin inducible; auxin maxima is localized at the tip of the developing primordia and several cell wall modelling enzymes (Cosgrove, 2000; Cosgrove, 2005) are auxin inducible in a LAX3 dependent manner. They proposed that auxin from the developing primordia acts as a signal to induce *LAX3* in the cortex. In a positive feedback loop, *LAX3* induction results in build-up of auxin in the cortex cells that then results in the induction of cell wall remodeling enzymes to facilitate smooth passage of the primordia through the cortex. Similar mechanism then facilitates primordia emergence through the epidermis. Porco et al. (2016a) provided further mechanistic insight into the regulation of lateral root emergence. They showed that induction of *LAX3* by auxin is mediated by *LBD16* which acts upstream of LAX3. Additionally, Orman-Ligeza et al. (2016) showed that reactive oxygen species (ROS) also involved in lateral root emergence by cell wall remodeling of overlaying tissues.

Light has been known to regulate root system architecture, but the mechanism has not been well understood. Recently, van Gelderen et al. (2018) revealed that light regulate lateral root development through regulating auxin transport. They showed that low R:FR perception in the shoot inhibits lateral root emergence. This is achieved *via* HY5 (ELONGATED HYPOCOTYL5) that accumulates in the lateral root primordia in phytochrome dependent fashion and regulates the plasma membrane abundance of LAX3 and PIN3 to reduce auxin levels in the overlaying cortex cells to reduce lateral root outgrowth.

### Adventitious Root Development

Adventitious roots are post embryonic roots and auxin has been known to regulate their formation (Veloccia et al., 2016; Fattorini et al., 2017). Recently, two independent reports suggest a possible role of AUX1 and LAX3 in this process. Veloccia et al. (2016) showed that both ethylene and auxin regulate adventitious root formation in Arabidopsis. Treatment of Arabidopsis seedlings with ethylene precursor ACC (1-aminocyclopropane-1-carboxylic acid) resulted in increased number of adventitious roots in AUX1/LAX3 dependent fashion. It appears that AUX1/LAX3 mediated adventitious root formation is regulated by ARF7/ARF19-LBD16/LBD18 module as aux1, lax3, lbd16, lbd18, arf7, and arf19 single mutants have reduced number of adventitious roots whereas aux1lax3lbd16lbd18 quadruple mutants lack adventitious roots (Lee et al., 2019).

### Root Hair Development

Auxin plays a critical role in root hair development (Knox et al., 2003; Fischer et al., 2006; Salazar-Henao et al., 2016). Auxin treatment promotes root hair elongation and mutations in AUX1 gene results in shorter root hairs that can be restored to wild type levels by exogenous auxin. Interestingly, AUX1 is expressed in non-hair epidermal cells but not in the hair cells (Jones et al., 2009). Computer simulation showed that expression of AUX1 in the non-hair cells can still result in over 10-fold accumulation of auxin in hair cells and thus Jones et al. (2009) concluded that non-hair cells affect auxin abundance in hair cells. PIN2 which is expressed in both root hair and non-hair cells can facilitate auxin efflux out of the non-hair cells and into the apoplast and despite no AUX1 in the hair cells, these root hair cells can still maintain high auxin concentration.

Root hair cell polarity has also been shown to be regulated by auxin (Grebe et al., 2002). Root hairs are formed on the basal side of the hair cells and auxin treatment results in more basal position of root hairs (Grebe et al., 2002). AUX1 has been implicated in regulating this root hair polarity by auxin as in aux1 mutants root hairs are formed not only on more apical side but they also have 30% increased frequency of double root hairs (Grebe et al., 2002).

Recently, Dindas et al. (2018) showed that AUX1 also mediates proton-coupled auxin transport in root hairs. Membrane depolarization is one of the earliest auxin responses in a cell. Using an electrophysiological approach and measuring membrane potential using intracellular mini-electrodes, Dindas et al. (2018) showed that IAA alters membrane potential in a pH and concentration dependent manner and aux1 mutants are severely impaired in IAA mediated membrane depolarization. They also showed that IAA influx is coupled with changes in cytosolic calcium and calcium influx is impaired in aux1 mutants. Interestingly, they find that auxin receptors TIR1/AFB are also involved in IAA mediated membrane depolarization and calcium influx. This suggests that early events in auxin signaling are non-genomic. This led Dindas et al. (2018) to propose a very short auxin signaling module, where a cytosolic component of SCFTIR/AFB binds to IAA that then regulates opening of calcium channels and hence an increase in intracellular Ca++ levels.

Auxin mediated root hair elongation is a key adaptive response to low P (Bates and Lynch, 1996; Lynch, 2011). Recently, Giri et al. (2018) and Bhosale et al. (2018) showed that root hair elongation under low P is mediated by AUX1. Bhosale et al. (2018) provided a mechanistic frame work for low P mediated root hair elongation in Arabidopsis. They showed that auxin homeostasis (Porco et al., 2016b) is crucial for root hair elongation under low P. They also showed that under low P, there is accumulation of auxin in the root apex through induction of TAA1, a key enzyme in auxin biosynthesis. AUX1 then facilitates the movement of this auxin in a shootward direction into the root hair zone where it facilitates root hair elongation. In this study, they not only showed that both taa1 and aux1 mutants have defects in root hair elongation under low P but also mapped the tissues required for root hair elongation under low P. They showed that expression of AUX1 in lateral root cap and epidermal cells files is sufficient to rescue low P mediated root hair elongation defect in aux1 mutants. But what happens when auxin has reached the root hair zone? What are the down-stream components mediating this low P mediated root hair elongation response? To investigate this, Bhosale et al. (2018) used a global gene expression profiling approach and showed that auxin response factor ARF19 is induced under low P and genetic analysis confirmed the role of ARF19 in regulating root hair elongation response under low P as arf19 mutants were defective in root hair elongation under low P. Next, they asked the question, what are the targets for ARF19? bHLH transcription factor RSL4 is a known regulator of root hair elongation and the transcriptome as well as reporter studies suggested that RSL4 and its close homolog RSL2 both are induced in the root apex under low P and both rsl4 and rsl2 mutants had defects in low P mediated root hair elongation. A close examination of the RSL2 and RSL4 promoters revealed several auxin response elements suggesting a possible mechanism for auxin mediated root hair elongation. Based on these results, Bhosale et al. (2018) proposed that low P results in increased auxin accumulation under low P through TAA1. This auxin is then moved to the root hair zone through AUX1 where it induces the expression of auxin inducible ARF19 that then induces the expression of bHLH transcription factors RSL2 and RSL4 that facilitate root hair elongation.

### Leaf Morphogenesis

PIN based auxin efflux has been previously shown to define regions of fast and slow growing areas in leaf margins regulated by CUC (CUP-SHAPED COTYLEDON) transcription factors, that are known to regulate organ boundaries in plants (Nikovics et al., 2006). Recently, Kasprzewska et al. (2015) have shown that auxin influx is also required for leaf serration in Arabidopsis. AUX1, LAX1, and LAX2 are all expressed in the leaves and show non-overlapping and dynamic expression patterns. AUX1 expression is more confined to the leaf margins. In contrast, LAX2 expression is excluded from the margins and is localized more towards the center of the leaf primordia and gradually gets confined to the leaf vasculature. LAX1 expression appears to be most dynamic and is mainly seen in the leaf tip and the flanks in the young leaf primordia. Later, new LAX1 expression sites are seen at the leaf margins proximal to the original sites which Kasprzewska et al. (2015) argued could be “presumptive sites of serration”. Despite non-overlapping expression patterns, single or double AUX1/LAX mutants don’t show any leaf serration defects. However, aux1/lax quadruple mutants and aux1lax1lax2 triple mutants have reduced leaf serrations. Kasprzewska et al. (2015) argued that this cannot be explained by simple genetic redundancy and so they used a modelling approach and proposed a “margin-patterning” model in which AUX1/LAX1/LAX2 auxin import module regulates extent of leaf serration.

More recently, Moreno-Piovano et al. (2017) also showed the involvement of LAX2 in leaf venation patterning and normal xylem development. They showed that lax2 mutants have increased xylem length and number of xylem cell rows which can be restored by expression of LAX2 suggesting that auxin homeostasis regulates leaf venation patterning.

### Female Gametophyte Development

Female gametophyte (megagametophyte) development begins with mega spore mother cell undergoing meiotic division and giving rise to four haploid cells. One of the haploid cell becomes a functional megaspore and undergoes three rounds of mitosis to produce seven-celled eight-nucleate highly polarized megagametophyte comprised of two synergid cells, one egg cell, one central cell and three antipodal cells. Auxin efflux carrier PIN1 was previously shown to play a role in regulating female gametophyte development (Ceccato et al., 2013). Recently, Panoli et al. (2015) showed that auxin influx and local auxin biosynthesis are also crucial in this process. They revealed that while AUX1 is primarily localized in the synergids and egg cell membranes, LAX1 is seen localized in the sporophytic tissues of nucellus surrounding the micropylar pole of embryo sac. Single, double and triple and quadruple mutants’ analysis of AUX1/LAX family members revealed that aux1lax1lax2 triple mutant and aux1lax1lax2lax3 quadruple mutant had multiple gametophyte defects with about 29% ovules showing aberrant embryo sacs. Panoli et al. (2015) further provided evidence that besides the auxin import, local auxin biosynthesis through YUCCA/TAA pathway also mediate mitotic cell division and cell specification during female gametophyte development.

### Embryo Development

Genetic and pharmacological studies show that auxin is crucial for embryo development (Hardtke and Berleth, 1998; Bhatia et al., 2016). For example, mutations in one of the key auxin signaling genes *MONOPTEROS/AUXIN RESPONSE FACTOR5* result in severe embryonic defects (Hardtke and Berleth, 1998; Bhatia et al., 2016). In addition, previous studies have shown the importance of auxin transport in embryo development as AUX1/LAX quadruple mutant are reported to have disorganized radicle apex and an increase in the root-cap cell numbers and/or cell size (Ugartechea-Chirino et al., 2010).

More recently, Robert et al. (2015) showed that auxin influx carriers AUX1, LAX1, and LAX2 are required for embryonic root and shoot pole formation. Of the four AUX1/LAX genes, AUX1, LAX1, and LAX2 are expressed in the embryo. They showed that AUX1 is specifically expressed in the 32-cell embryo stage and later in the provascular cells. LAX2 is also expressed in the perivascular cells from 32-cell embryo stage onward and is also expressed in the hypophysis and the uppermost suspensor cell. In contrast, LAX1 is expressed very early on from the one-cell stage in the apical cell and from 32-cell stage, its expression is more pronounced in the upper tier cells and by heart stage embryo, LAX1 expression is confined to the cotyledon tips. No LAX3 expression is reported in the embryo.

To get a better understanding of the role of auxin influx carriers in embryo development, Robert et al. (2015) specifically used AUX1/LAX double and triple mutant combinations as previous studies had reported no embryo related defects in aux1, lax1, and lax2 single mutants (Bainbridge et al., 2008). They uncovered patterning defects in the upper pole in the aux1lax1 double mutants at a low frequency and this was considerably more pronounced both in the severity and the frequency in the aux1lax1lax2 triple mutants. The embryo defects were manifested later in the seedlings as about one quarter of the seedlings showing mono cotyledon and/or stubby roots. These defects resembled monopteros (mp) and bodenlos (bdl) mutants (Berleth and Jürgens, 1993; Hardtke and Berleth, 1998; Hamann et al., 1999). Furthermore, they observed that expression of AUX1 and LAX2 but not LAX1 was reduced or absent in the strong MP allele mpB4149. This led Robert et al. (2015) to conclude that MP/BDL signaling module regulates AUX1/LAX mediated auxin import into the cell.

More recently, Liu et al. (2017) showed that ROPGEF1 regulates AUX1 polar localization in the embryo and the roots. ROPGEFs are guanine nucleotide exchange factors that are known to activate Rho GTPases of plants. AUX1 is localized to the apical face of the cell in the embryo central vascular cells and the protophloem cells. Liu et al. (2017) showed that in ropgef1 mutant apical AUX1 localization has shifted from apical to basal position in the embryo and also in the root protophloem cells. They also showed that ropgef1 mutants also have altered accumulation of PIN2 and PIN7 and cannot establish asymmetric auxin gradient in gravistimulated roots and have embryo defects as well as cotyledon vein breaks and altered root gravitropic response.

## AUX1/LAX Gene Family and Their Roles Across Plant Species

**Figure 2:**
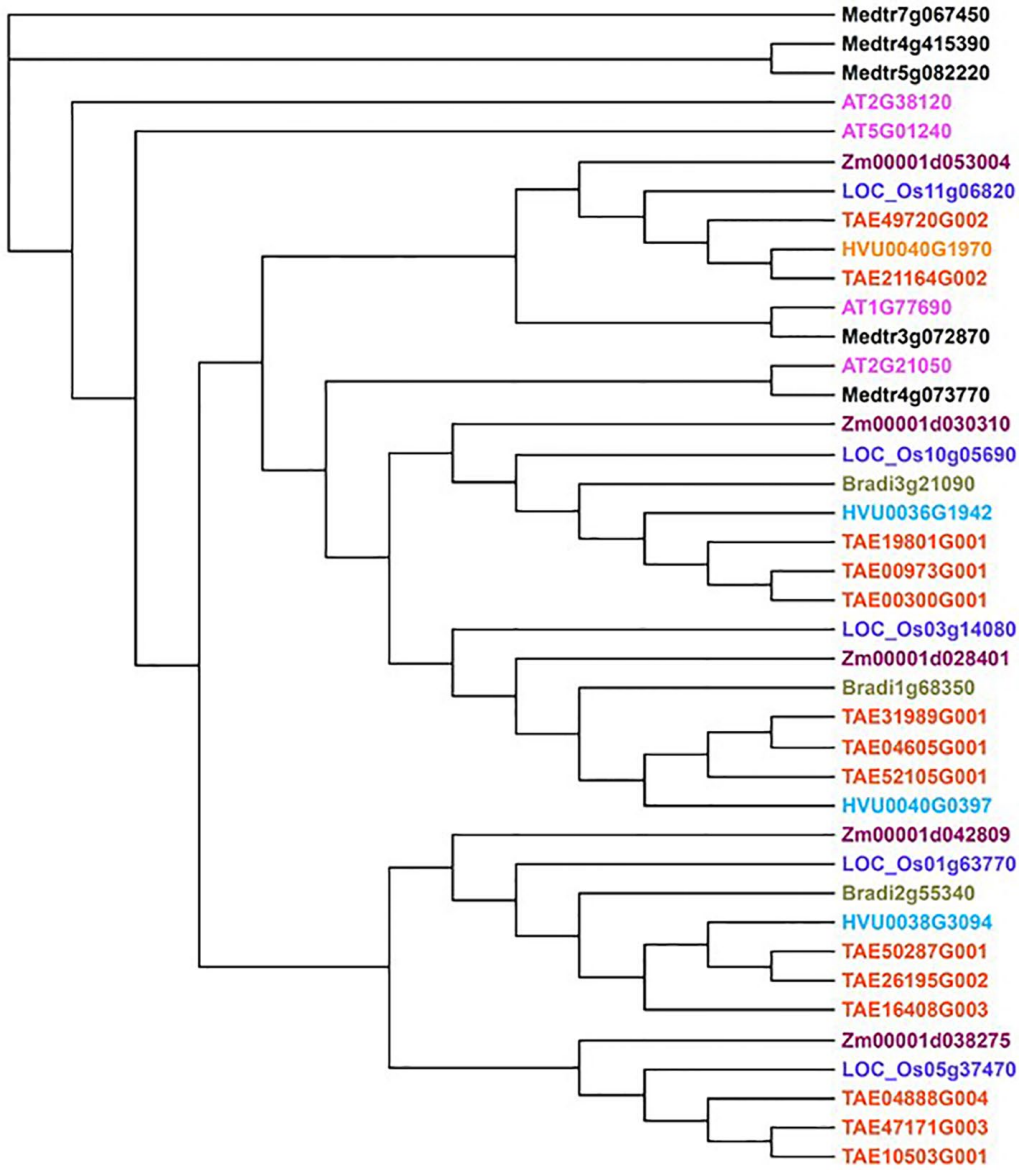
The phylogeny of *AUX1* homologs of selected plant species. This tree was generated using interactive phylogenetic module from Plaza 4.0 (Van Bel et al., 2017). *AtAUX1* was used as seed gene to retrieve homologs of selected species [Arabisdopsis thaliana (AT), Zea maize (Zm), Oryza sativa ssp. Japonica (LOC), Triticum aestivum (TAE), Hordeum vulgare (HVU), Brachypodium distachyon (Bradi) and Medicago truncatula (Medtr); coloured differently] and homologs with >70% protein identity were retained for generating the tree. Some of these homologs have been previously characterised (discussed in the main text).

**Figure 3:**
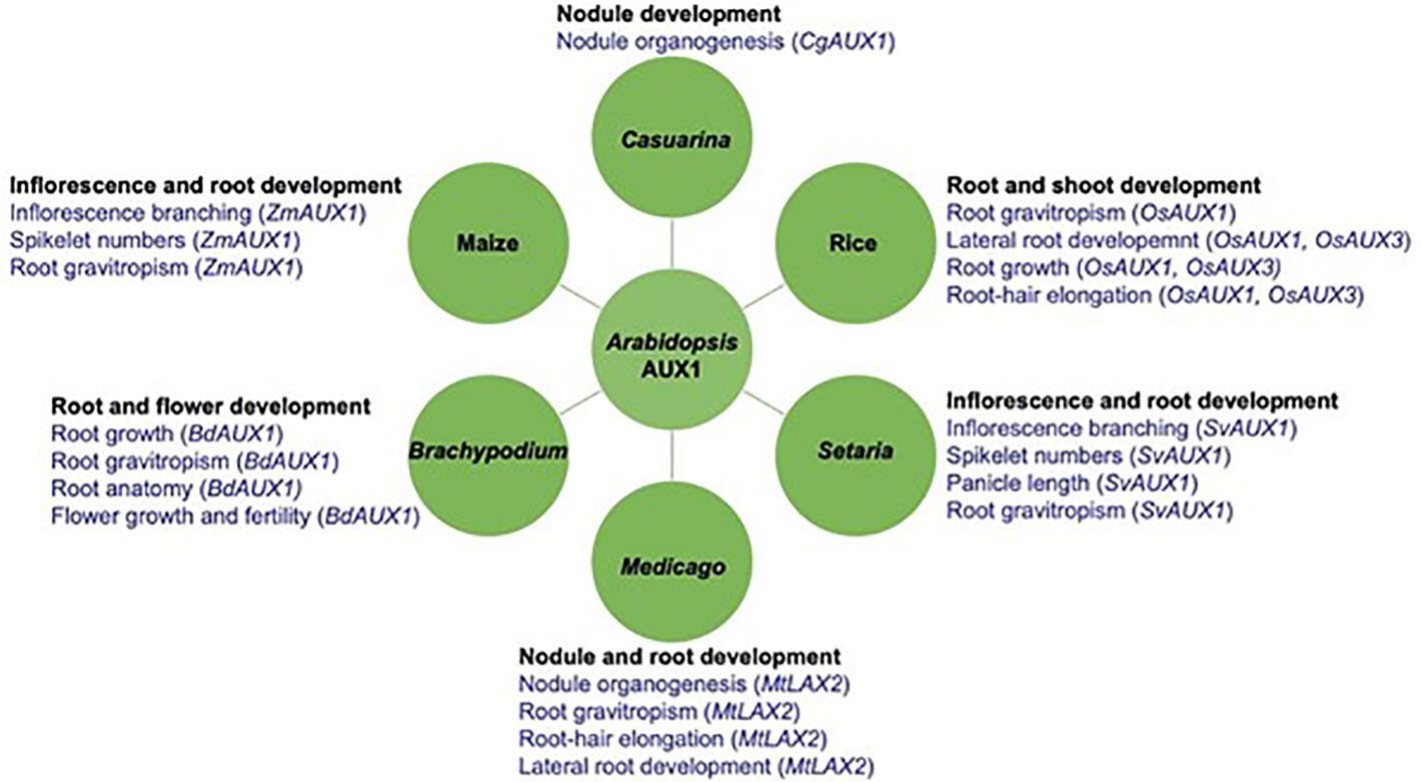
*Arabidopsis AUX1* homoliogs play crucial roles in plant development across severals species. Arabidopsis auxin influx carrier *AUX1* homologs regulate aspects of plant development such as root gravitropism; root architecture (e.g. root/lateral root development; root hair development); root natomy (e.g. cortical cell number and size); inflorescence architecture (e.g. branching; spikelet numbers; panicle lenght); nodule organogenesis and flower development in several plants including the one represented here: Rice, Maize, *Setaria*, *Brachypodium, Medicago*, and *Casuarina*. Names in parenthesis indicate the genes controlling the respective role.

With advances in genome sequencing, *AUX1/LAX* homologs have been identified in several plant species (**Figure 2**) including rice, maize, wheat, barley, *Setaria, Medicago*, and *Brachypodium*. (Hochholdinger et al., 2000; Zhao et al., 2015;Huang et al., 2017; van der Schuren et al., 2018). They all show high similarity to AtAUX1 at protein level (71–90%). In this section, we will briefly review our current understanding of the role of *AUX1/LAX* gene family across plant species. For sake of brevity, we will only focus on model plants where there are supporting functional and/or genetic evidence for the role of *AUX1/LAX* genes in regulating plant development (**Figure 3**).

### Inflorescence Architecture in *Setaria* and Maize

Recently, Huang et al. (2017) demonstrated that *AUX1* homologs in *Setaria viridis* (*SvAUX1) *and maize (*ZmAUX1*) are involved in inflorescence development and root gravitropism. Inflorescence architecture is an important agronomic trait as it influences grain yield. Using a forward genetic approach, Huang et al. (2017) showed that in *S. viridis*, mutations in *SvAUX1*, result in major defects in inflorescence branch development leading to sparse panicle (spp) phenotypes. These mutants (*spp1-1* and *spp1-3*) also show decreased plant height, reduced inflorescence branching and spikelet numbers and increased panicle length compared to the control plants.

In maize, auxin synthesis, transport and signaling have been previously linked to inflorescence architecture variation, including branching pattern changes (Gallavotti et al., 2008; Skirpan et al., 2009; Phillips et al., 2011). Auxin efflux carrier *ZmPIN1* has been also implicated in maize inflorescence development (Skirpan et al., 2009). Now Huang et al. (2017) showed that a loss-of-function allele of *ZmAUX1* termed *Zmaux1-0* has reduced inflorescence branching and fewer spikelets in the central spike. Interestingly, Huang et al. (2017) observed that these defects in inflorescence development in *Zmaux1-0* are less severe than previously studied auxin synthesis mutants *sparse inflorescence1* and *vanishing tassel1* and auxin transport mutant *bif2* suggesting potential redundancy among *ZmAUX1* and its other three homologues that show overlapping expression in immature inflorescence.

Additionally, Huang et al. (2017) observed that *spp-1*, *spp1-3*, and *Zmaux1-0* mutants have root agravitropic defects similar to *ataux1*. But unlike *ataux1*, *spp-1* and *spp1-3* mutants have no lateral root defects.

### Root and Shoot Development in Rice

Like *Arabidopsis*, *OsAUX1* has been shown to regulate root gravitropism and lateral root development (Zhao et al., 2015) and low P mediated root hair elongation (Giri et al., 2018). Zhao et al. (2015) observed that *OsAUX1* is highly expressed in lateral roots and lateral root primordia. Mutations in *OsAUX1* result in reduced lateral root initiation events whereas *OsAUX1* overexpression plants exhibit increased lateral root initiation events. Transcript levels of several auxin signaling and cell cycle genes are significantly downregulated in *osaux1*, further highlighting the importance of OsAUX1 in regulating lateral root development in rice.

*OsAUX1* has also been implicated in Cadmium (Cd) stress response. Cd stress induces the production of reactive oxygen species, which trigger cell death in plants. Auxin signaling is known to be involved in activating Cd-induced morphogenic defense responses in wheat, barley and *Arabidopsis* (Tamas et al., 2015; Agami and Mohamed, 2013; Zhu et al., 2013). Yu et al. (2015) showed that *OsAUX1/LAX* genes (*OsAUX1-5*) are induced by Cd stress. Reporter analysis showed that *OsAUX1* is distinctly induced under Cd stress in primary roots, lateral roots and root hairs and *osaux1* mutants are more sensitive to Cd stress. Cd contents in the *osaux1* mutant were not altered, but reactive oxygen species-mediated damage was enhanced, further increasing the sensitivity of the mutant to Cd stress. Taken together, their results indicated that OsAUX1 plays an important role in mediating plant responses to Cd stress. Yu et al. (2015) also showed that in contrast to *Arabidopsis*, *OsAUX1* is specifically expressed in root hair cell files, suggesting functional differences between monocots and dicots in regulating RH development.

Giri et al. (2018) showed that OsAUX1 also regulates root hair elongation under low P as osaux1 mutants are defective in low P mediated root hair elongation. Using direct auxin quantification by mass spectrometry as well as auxin reporter-based approaches, they showed that low P results in increased auxin accumulation in the root apex in OsAUX1 dependent fashion. Although mechanistic details are not yet fully understood in rice, it is tempting to speculate that it is similar to Arabidopsis (Bhosale et al., 2018) and may even be conserved in other land plants as well. Further, using an elegant split root experiments, by exposing half of the crown roots from the same plants to low P and the other half to high P, Giri et al. (2018) also showed that low P mediated root hair elongation is a local response irrespective of the plant P status.

More recently, Wang et al. (2019) characterized another AUX1 homolog in rice OsAUX3. OsAUX3 is expressed in primary roots, lateral roots and in the root hairs. Mutations in OsAUX3 result in shorter primary roots, decreased lateral root density, and longer root hairs compared to control. In addition, it appears that OsAUX3 is also involved in mediating Aluminum (Al) induced inhibition of root growth. OsAUX3 expression is up-regulated in the root apex under Al stress and one of the OsAUX3 mutants (osaux3‐2) is insensitive to Al treatments and Al-induced ROS mediated damage.

### Root and Flower Development in *Brachypodium*

Recently, van der Schuren et al. (2018) showed that similar to *AtAUX1, BdAUX1* is expressed in roots displaying expression patterns in root protophloem, epidermis and columella. Additionally, authors observed that unlike *AtAUX1*, *BdAUX1* is also expressed throughout the stele and in the outer cortex layers, encompassing the combined expression domains of *AtAUX1*, *AtLAX2*, and *AtLAX3*. Thus, possibly *BdAUX1* have the combined functions in these tissues. *Bdaux1* mutant roots are agravitropic and show longer root phenotypes due to increased mature cell lengths possibly due to higher free auxin content. Additionally, *Bdaux1* mutants are also significantly thinner due to reduced cell file numbers in every tissue except xylem and phloem and due to smaller cell sizes radially. *Bdaux1* mutants also show reduced root hair length and density. Unlike *AtAUX1*, *Bdaux1* loss‐of‐function mutants are dwarfs with aberrant flower development, and consequently infertile suggesting a more crucial role for BdAUX1 in flower development.

### Nodule Organogenesis in *Medicago* and *Casuarina*

Péret et al. (2007) have previously shown that nodule organogenesis in *Casuarina glauca* is facilitated by CgAUX1. More recently, Roy et al. (2017) showed that nodule organogenesis in *Medicago* is mediated by MtLAX2. They showed that *MtLAX2* is auxin inducible and is expressed in the nodule primordia, vasculature of developing nodules and at the apex of matured nodules. Upon Rhizobium infection, *mtlax2* mutants have fewer nodules and reduced DR5 activity at the infection sites clearly implicating the role of *MtLAX2* in nodule development.

In addition, Péret et al. (2007) also showed the importance of MtLAX2 in root development. Mutation in *MtLAX2* results in defects in root gravitropism, fewer lateral roots and shorter root hairs suggesting this to be a functional analog of *AtAUX1* (Roy et al., 2017). Interestingly, *MtLAX2* cannot rescue root gravitropic defects of Arabidopsis *aux1* mutant when expressed under *AtAUX1* promoter. This led Roy et al. (2017) to conclude that these genes may have diverged to an extent that they encode biochemically distinct proteins. It is possible that MtLAX2 also cannot get correctly localized in AUX1 expression domain as has been the case for AtLAX2 and AtLAX3 (Péret et al., 2012). Modeling studies on auxin transport.

## Modeling Studies on Auxin Transport

Computer simulations and modelling approaches in the past decade have proven very useful in getting better understanding of the role of auxin transport in regulating auxin mediated developmental processes in *Arabidopsis* and how auxin fluxes are established and maintained (Swarup et al., 2005; Kramer and Bennett, 2006; Grieneisen et al., 2007; Laskowski et al., 2008; Jones et al., 2009; Prusinkiewicz et al., 2009; Mironova et al., 2010; Szymanowska-Pułka and Nakielski, 2010; Vernoux et al., 2011; Bridge et al., 2012; Steinacher et al., 2012; Novoselova et al., 2013). Increased understanding of the auxin transport proteins and their sub-cellular localization have helped refine previous auxin-transport models and improved our understanding of how changes at cellular level regulate organ-scale auxin patterns. For example, Band et al. (2014) showed the importance of AUX1/LAX proteins in pattern formation at the root tip by taking into consideration the localization of auxin transport proteins as well as cell geometries and further validated their model predictions using DII VENUS auxin sensor (Brunoud et al., 2012). Authors found that, while polar localized auxin efflux carriers provide polarity of the auxin movement, nonpolar AUX1/LAX influx carriers are crucial in determining which tissues have high auxin levels. They concluded that both auxin influx and efflux carriers are required to create a pattern of auxin distribution in the root tip.

More recently, Moore et al. (2017) developed a mechanistic model that also reinforced that auxin pattern formation requires co-ordination between influx and efflux carriers. Their model predicts that the localization of influx carriers can either get more polar when auxin efflux carrier levels are changed or modulate efflux carrier level and polarity to maintain the auxin patterns.

## Conclusion and Perspectives

In the past two decades, there has been a significant increase in our understanding of molecular basis of auxin transport and roles of auxin transporters in plant development. Particularity, there has been a better understanding of auxin influx carriers and how they play crucial roles in almost all aspects of plant growth and development. More advanced computer models and high-resolution imaging and segmentation approaches have proved crucial in providing better understanding of auxin influx carriers in pattern formation especially how changes at the cellular scale affect organ-scale auxin patterns.

Root development is very plastic and respond to their environment. Recently it has been shown that chromium inhibits primary root growth by regulating cell cycle genes. Chromium toxicity can cause major damage to crop yield. Genetic and physiological studies show a role for AUX1 in chromium mediated inhibition of root growth (Wakeel et al., 2018). Similarly, as stated above, AUX1 has also been implicated in Al and Cd mediated inhibition of root growth. Similarly, low P mediated root hair elongation response is mediated *via* AUX1 but early events are not well understood as to how low P status is sensed by the plants. Further understanding of the early events will be crucial for our understanding of the root growth and development in changing environment and may help develop predictive models for future crop improvement programmes.

Alternative splicing is another key area that has not been explored much in the plants but may be crucial for better understanding of the role of alternatively spliced transcripts in regulating and shaping plant development. It appears that alternative splicing in plants is more common than previously appreciated (Li et al., 2016; Swarup et al., 2016). With the advancement in sequencing technology, longer reads and single cell transcriptome, it is possible now to get a much better view of the cellular transcriptome and what if any is the role of alternative spliced transcripts in regulating auxin transport. 

## Author Contributions

RS and RB did the review of literature, prepared figures and wrote the manuscript.

## Conflict of Interest

The authors declare that the research was conducted in the absence of any commercial or financial relationships that could be construed as a potential conflict of interest.
